# Inhibition of integrin β1-mediated oncogenic signalling by the antitumor *microRNA-29* family in head and neck squamous cell carcinoma

**DOI:** 10.18632/oncotarget.23194

**Published:** 2017-12-11

**Authors:** Keiichi Koshizuka, Naoko Kikkawa, Toyoyuki Hanazawa, Yasutaka Yamada, Atsushi Okato, Takayuki Arai, Koji Katada, Yoshitaka Okamoto, Naohiko Seki

**Affiliations:** ^1^ Department of Functional Genomics, Chiba University Graduate School of Medicine, Chuo-ku, Chiba, Japan; ^2^ Department of Otorhinolaryngology/Head and Neck Surgery, Chiba University Graduate School of Medicine, Chiba, Japan

**Keywords:** microRNA, miR-29a, miR-29b, miR-29c, ITGB1

## Abstract

Due to their aggressive behavior, local recurrence and distant metastasis, survival rate of advanced stage of the patients with head and neck squamous cell carcinoma (HNSCC) is very poor. Currently available epidermal growth factor receptor (EGFR)-targeted therapies are not considered curative for HNSCC. Therefore, novel approaches for identification of therapeutic targets in HNSCC are needed. All members of the *miRNA-29* family (*miR-29a*, *miR-29b*, and *miR-29c*) were downregulated in HNSCC tissues by analysis of RNA-sequencing based microRNA (miRNA) expression signature. Ectopic expression of mature miRNAs demonstrated that the *miR-29* family inhibited cancer cell migration and invasion by HNSCC cell lines. Comprehensive gene expression studies and *in silico* database analyses were revealed that integrin β1 (*ITGB1*) was regulated by the *miR-29* family in HNSCC cells. Overexpression of *ITGB1* was confirmed in HNSCC specimens, and high expression of ITGB1 significantly predicted poor survival in patients with HNSCC (*p* = 0.00463). Knockdown of *ITGB1* significantly inhibited cancer cell migration and invasion through regulating downstream of *ITGB1*-mediated oncogenic signalling. In conclusion, regulation of the antitumor *miR-29* family affected integrin-mediated oncogenic signalling to modulate HNSCC pathogenesis; these molecules may be novel therapeutic targets for HNSCC.

## INTRODUCTION

Patients with head and neck squamous cell carcinoma (HNSCC) usually have metastatic lesions when the disease is first diagnosed, and the 5-year survival rate is less than 50% [1, 2]. The epidermal growth factor receptor (EGFR)-targeting monoclonal antibody cetuximab has been approved for the treatment of patients with HNSCC, the curative effects of this drug are limited [3, 4]. Therefore, the identification of novel molecular targets in metastatic activating pathways would help to develop new therapies for this disease.

MicroRNAs (miRNAs) are small, noncoding RNAs that function to fine-tune the expression of protein-coding or protein-noncoding RNAs by sequence-dependent manner [5, 6]. Accumulating evidence has showed that aberrant expression of miRNAs disrupts systematically controlled RNA networks in cancer cells [7]. These events are deeply involved in cancer progression, metastasis, and drug resistance [8]. We have sequentially identified novel cancer pathways based on antitumor miRNAs in several cancers [9–14].

Analysis of our miRNA expression signatures based on RNA-sequencing showed that *miR-29* family members (i.e., *miR-29a*, *miR-29b*, and *miR-29c*) are frequently detected as downregulated miRNAs in several types of cancers [10, 15–17]. Moreover, functional studies have revealed that all members of the *miR-29* family inhibit cancer cell migration and invasion, suggesting that these miRNAs are involved in metastatic pathways in human cancers [11, 18, 19]. Our previous studies have shown that restoration of the *miR-29* family significantly inhibits cancer cell aggressiveness through targeting lysyl oxidase like-2 (*LOXL2*) in HNSCC, renal cell carcinoma, and lung cancer [11, 20, 21]. *LOXL2* functions is crosslinking of collagen and elastin in the extracellular matrix (ECM), and overexpression of *LOXL2* has involved in human pathogenesis, fibrosis and cancers [22]. Knockdown of *LOXL2* significantly inhibits cancer cell migration and invasion, indicating that *LOXL2* could be a new therapeutic target in cancers.

In HNSCC, several studies have demonstrated the downregulation of the *miR-29* family and their antitumor functions through targeting several oncogenic genes [19, 21]. We previously found that downregulation of *miR-29* family and overexpression of laminin (*LAMC2*) and integrin (*ITGA6*) in HNSCC cells, thereby activating cancer cell migration and invasion [19]. Several studies have demonstrated that activation of ECM/integrin-mediated signalling contributes to cancer cell progression and metastasis [23, 24]. Thus, we hypothesised that identification of cancer networks mediated by the antitumor *miR-29* family may provide novel insights into the therapeutic targets of HNSCC. In this study, we conducted re-analysis of therapeutic targets regulated by the antitumor *miR-29* family in HNSCC cells and identified two key molecules, *ITGA6* and *ITGB1*. Regulation of *ITGA6* and the *miR-29* family has been reported previously [19]. Thus, we focused on *ITGB1* and investigated the functional significance of the gene in HNSCC pathogenesis.

## RESULTS

### Expression levels of *miR-29* family members in HNSCC clinical specimens and cell lines

The expression levels of *miR-29a*, *miR-29b*, and *miR-29c* were significantly lower in cancer tissues (n = 22) and HNSCC cell lines (SAS and HSC3) than in normal epithelial tissues (*n* = 22) (*P* < 0.0001, *P* = 0.0003, and *P* < 0.0001, respectively; Supplementary Figure 1A).

The *miR-29* family members were clustered at two different human chromosome loci (*miR-29b-1* and *miR-29a* at 7q32.3; *miR-29b-2* and *miR-29c* at 1q32.2). Spearman’s rank test showed positive correlations between the expression of *miR-29a* and *miR-29b* (R = 0.648 and *P* < 0.0001) and between *miR-29b* and *miR-29c* (R = 0.746 and *P* < 0.0001; Supplementary Figure 1B).

### Identification of target genes regulated by the *miR-29* family in HNSCC cells

**Figure 1:**
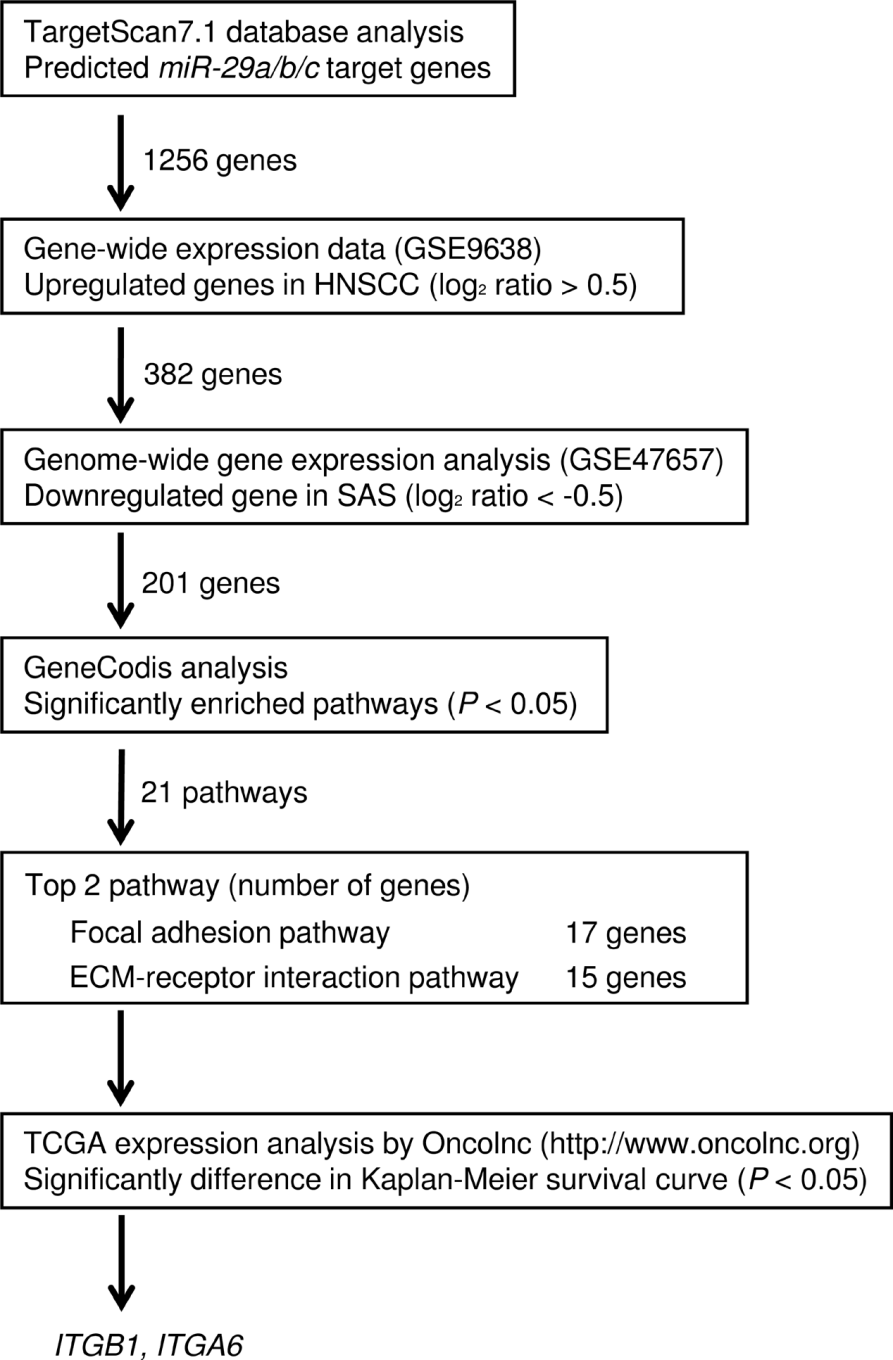
Flow chart depicting the strategy for identification of putative target genes regulated by the *miR-29* family in HNSCC cells

Our strategy for selection of genes regulated by the *miR-29* family is shown in Figure 1. The TargetScan7.1 database showed that 1,256 genes had putative target sites for the *miR-29* family in their 3′UTRs. First, to evaluate upregulated genes in clinical HNSCC specimens (accession number: GSE9638). A total of 382 genes were selected as putative targets for the *miR-29* family in HNSCC cells (log_2_ ratio > 0.5). Next, we performed comprehensive gene expression analyses using SAS transfectant (GEO accession number: GSE47657). Genes downregulated (log_2_ ratio < -0.5) by transfection with *miR-29a* were merged with TargetScan-selected genes. A total of 201 genes were downregulated in *miR-29a* transfectants and had putative binding sites in their 3′UTRs, respectively.

Furthermore, we categorised these putative targets into KEGG pathways using the GeneCodis database. A total of 21 pathways were identified as *miR-29* family regulated pathways (Table 1). Among these pathways, we focused on the “Focal adhesion pathway” and “ECM-receptor interaction pathway” because the *miR-29* family markedly inhibited cancer cell migration and invasion abilities [19]. A total of 17 genes were identified from these two pathways (Tables 2 and 3).

**Table 1:** Significantly enriched pathways regulated by the miR-29 family in HNSCC cells

Number of genes	Annotations	*P*-value
17	(KEGG) 04510: Focal adhesion	1.0E-13
15	(KEGG) 04512: ECM-receptor interaction	1.9E-16
14	(KEGG) 04974: Protein digestion and absorption	1.3E-15
11	(KEGG) 05146: Amoebiasis	5.6E-10
11	(KEGG) 05200: Pathways in cancer	5.9E-05
8	(KEGG) 05222: Small cell lung cancer	6.8E-07
6	(KEGG) 05410: Hypertrophic cardiomyopathy (HCM)	1.2E-04
6	(KEGG) 04010: MAPK signaling pathway	2.9E-02
5	(KEGG) 05414: Dilated cardiomyopathy	1.9E-03
5	(KEGG) 04514: Cell adhesion molecules (CAMs)	8.1E-03
4	(KEGG) 04930: Type II diabetes mellitus	1.8E-03
4	(KEGG) 05412: Arrhythmogenic right ventricular cardiomyopathy (ARVC)	7.7E-03
4	(KEGG) 04670: Leukocyte transendothelial migration	3.0E-02
4	(KEGG) 05145: Toxoplasmosis	3.5E-02
4	(KEGG) 04360: Axon guidance	3.8E-02
4	(KEGG) 04910: Insulin signaling pathway	3.9E-02
3	(KEGG) 00561: Glycerolipid metabolism	2.1E-02
3	(KEGG) 05130: Pathogenic Escherichia coli infection	2.8E-02
3	(KEGG) 04920: Adipocytokine signaling pathway	3.7E-02
3	(KEGG) 04260: Cardiac muscle contraction	4.7E-02
3	(KEGG) 00564: Glycerophospholipid metabolism	5.0E-02

**Table 2:** Focal adhesion pathway-related genes in HNSCC cells

Gene Symbol	Gene Name	conserved	poorly conserved	GSE9638 log_2_ ratio	GSE47657 log_2_ ratio
*ITGA6*	integrin, alpha 6	1	0	1.105	−1.620
*ITGB1*	integrin, beta 1 (fibronectin receptor, beta polypeptide, antigen CD29 includes MDF2, MSK12)	1	0	0.708	−0.566
*LAMC1*	laminin, gamma 1 (formerly LAMB2)	1	0	0.657	−1.711
*MAPK10*	mitogen-activated protein kinase 10	1	1	1.224	−0.909
*PDGFRB*	platelet-derived growth factor receptor, beta polypeptide	1	0	0.591	−0.738
*COL1A2*	collagen, type I, alpha 2	2	0	1.854	−0.606
*COL3A1*	collagen, type III, alpha 1	2	0	2.653	−0.782
*COL4A1*	collagen, type IV, alpha 1	2	0	1.932	−1.779
*COL4A2*	collagen, type IV, alpha 2	1	0	1.792	−1.757
*COL4A5*	collagen, type IV, alpha 5	2	0	1.364	−0.959
*COL4A6*	collagen, type IV, alpha 6	1	0	3.010	−0.870
*COL5A1*	collagen, type V, alpha 1	3	1	2.438	−1.561
*COL5A2*	collagen, type V, alpha 2	2	0	2.810	−3.182
*COL5A3*	collagen, type V, alpha 3	3	0	1.373	−0.701
*COL6A3*	collagen, type VI, alpha 3	1	0	1.782	−1.156
*COL6A6*	collagen, type VI, alpha 6	1	0	1.544	−0.741
*COL11A1*	collagen, type XI, alpha 1	2	0	1.038	−0.753

**Table 3:** ECM-receptor interaction pathway-related genes in HNSCC cells

Gene Symbol	Gene Name	conserved	poorly conserved	GSE9638 log_2_ ratio	GSE47657 log_2_ ratio
*ITGA6*	integrin, alpha 6	1	0	1.105	−1.620
*ITGB1*	integrin, beta 1 (fibronectin receptor, beta polypeptide, antigen CD29 includes MDF2, MSK12)	1	0	0.708	−0.566
*LAMC1*	laminin, gamma 1 (formerly LAMB2)	1	0	0.657	−1.711
*COL1A2*	collagen, type I, alpha 2	2	0	1.854	−0.606
*COL3A1*	collagen, type III, alpha 1	2	0	2.653	−0.782
*COL4A1*	collagen, type IV, alpha 1	2	0	1.932	−1.779
*COL4A2*	collagen, type IV, alpha 2	1	0	1.792	−1.757
*COL4A5*	collagen, type IV, alpha 5	2	0	1.364	−0.959
*COL4A6*	collagen, type IV, alpha 6	1	0	3.010	−0.870
*COL5A1*	collagen, type V, alpha 1	3	1	2.438	−1.561
*COL5A2*	collagen, type V, alpha 2	2	0	2.810	−3.182
*COL5A3*	collagen, type V, alpha 3	3	0	1.373	−0.701
*COL6A3*	collagen, type VI, alpha 3	1	0	1.782	−1.156
*COL6A6*	collagen, type VI, alpha 6	1	0	1.544	−0.741
*COL11A1*	collagen, type XI, alpha 1	2	0	1.038	−0.753

### Analysis of the clinical features of *ITGB1* in patients with HNSCC using TCGA database

**Figure 2:**
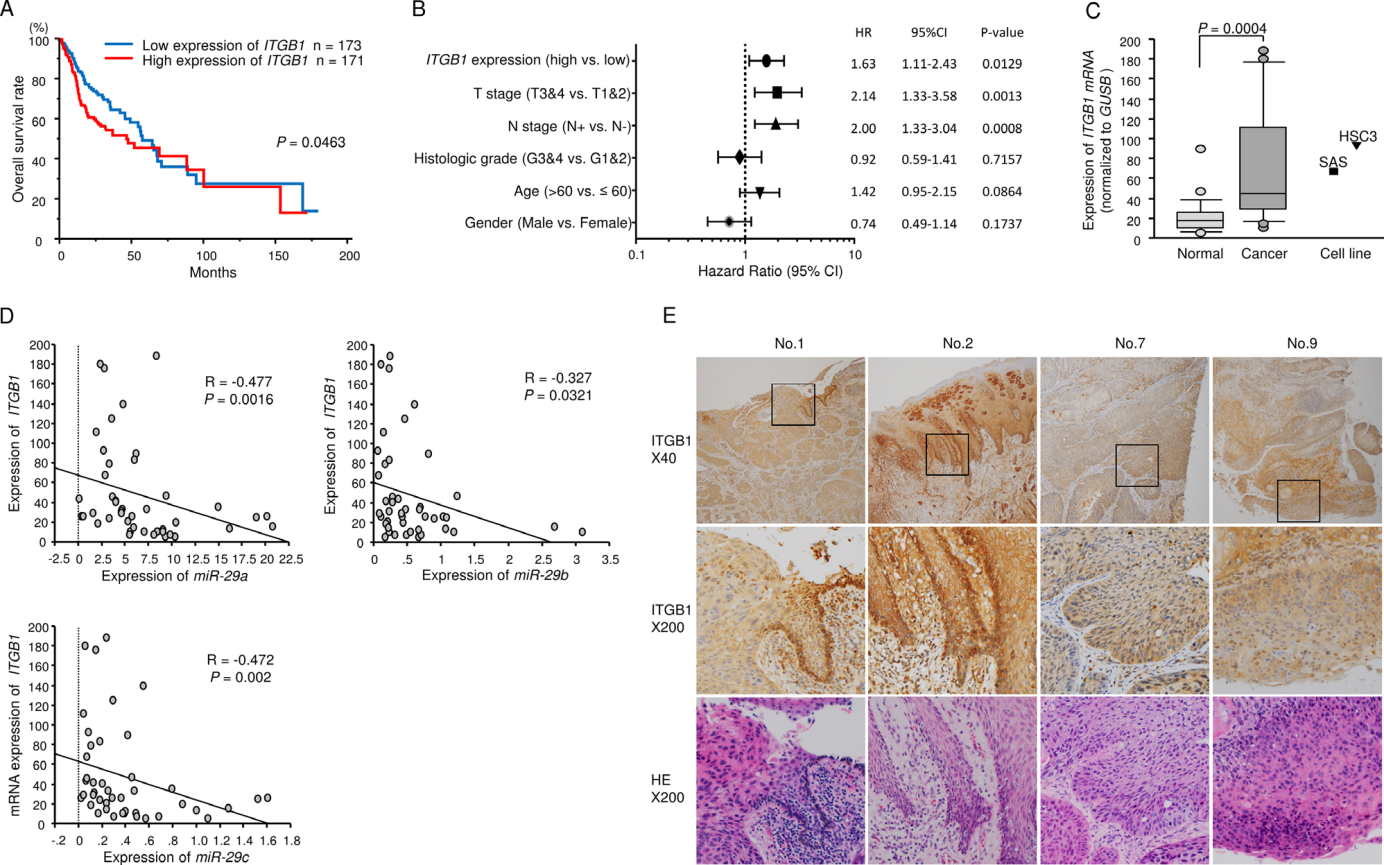
Expression of *ITGB1* and associations between *ITGB1* expression levels and clinical parameters in patients with HNSCC (**A**) Kaplan-Meier analysis of *ITGB1* expression and overall survival. (**B**) Multivariate Cox proportional hazards models. (**C**) Expression levels of *ITGB1* in HNSCC clinical specimens and cell lines. *GUSB* was used as an internal control. (**D**) The negative correlations between *ITGB1* expression and the expression of *miR-29a*, *miR-29b*, and *miR-29c*. Spearman’s rank test was used to evaluate the correlation. (**E**) Immunohistochemical staining of ITGB1 in HNSCC clinical specimens. The patients’ backgrounds and clinicopathological characteristics are summarised in Table 4. Primary rabbit anti-ITGB1 antibodies were diluted 1:100. The slides were treated with biotinylated goat anti-rabbit antibodies. ITGB1 was strongly expressed in cancer lesions (40× and 200× magnification).

We created survival curves of 17 putative target genes which were involved in “Focal adhesion pathway” and “ECM-receptor interaction pathway” using OncoLnc from TCGA database. We assessed the Kaplan–Meier univariate survival of patient groups using TCGA database; high expression of *ITGA6* and *ITGB1* was significantly associated with poor prognosis of patients with HNSCC (Figure 2A, Supplementary Figures 2A, 2B, and 3). The gene, *COL6A6*, showed the opposite behavior, its low expression was significantly associated with poor prognosis of the disease (*p* = 0.000993). Thus, we omitted it from future analysis.

We previously published several reports on *ITGA6* [15, 19]; therefore, in this study, we focused on *ITGB1* as a target gene of the *miR-29* family. Multivariate Cox proportional hazards models were used to assess independent predictors of progression-free survival. High *ITGB1* expression was a significant prognostic factor in patients with HNSCC (hazard ratio [HR] = 1.63, 95% confidence interval [CI] = 1.11–2.43, *P* = 0.0129; Figure 2B).

### Expression of *ITGB1*/ITGB1 in HNSCC clinical specimens

Expression of *ITGB1* was significantly upregulated in HNSCC tumor tissues (*P* = 0.0004, Figures 2C). Spearman’s rank test showed a negative correlation between the expression of *ITGB1* and *miR-29a* (*P* = 0.0016, R = -0.477), *ITGB1* and *miR-29b* (*P* = 0.0321, R = -0.327), and *ITGB1* and *miR-29c* (*P* = 0.002, R = -0.472; Figure 2D).

We also examined the expression levels of ITGB1 in HNSCC clinical specimens by immunohistochemistry. ITGB1 was strongly expressed in several cancer tissues (Figure 2E).

### *ITGB1* was directly regulated by the *miR-29* family in HNSCC cells

**Figure 3:**
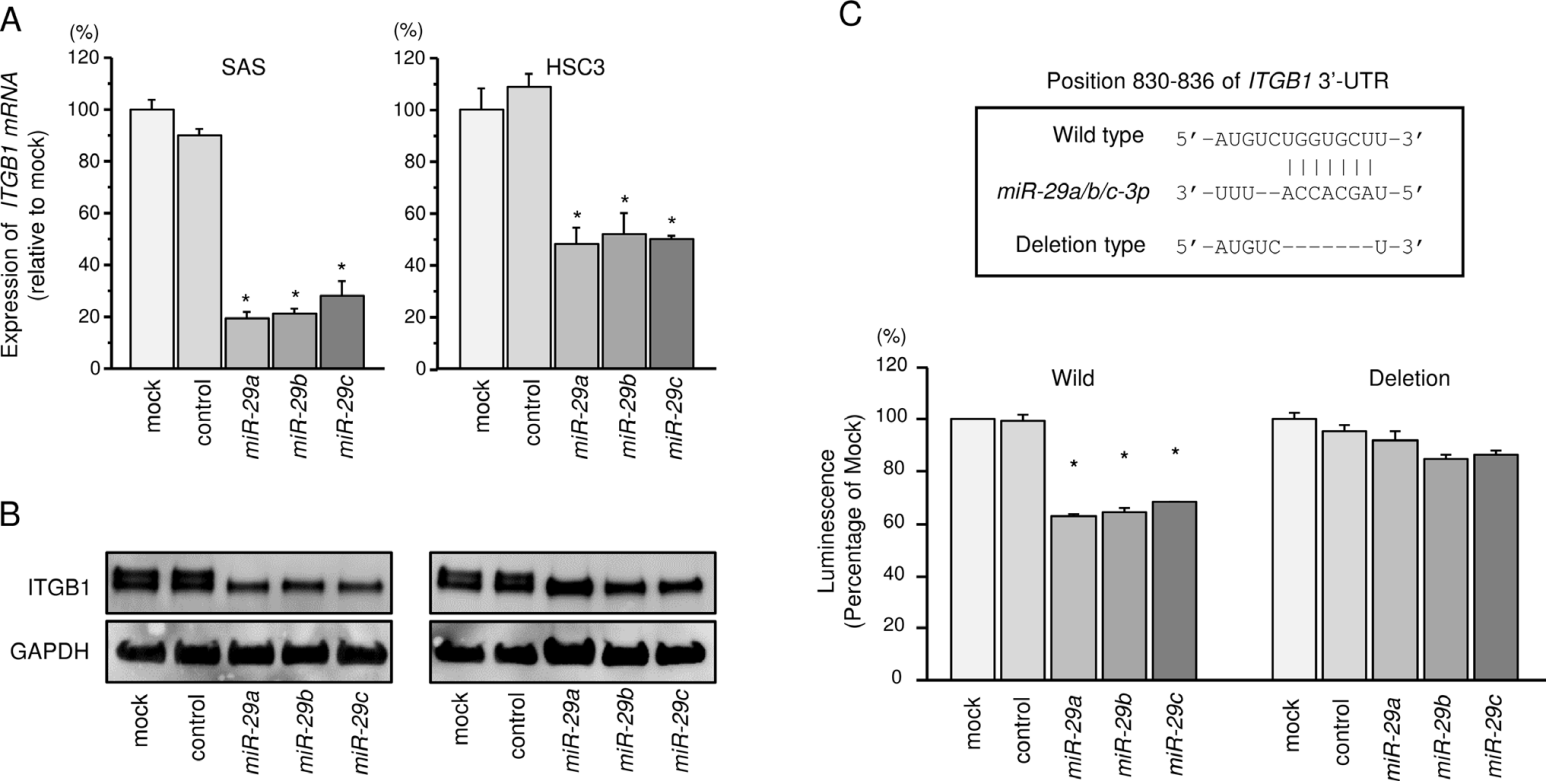
Regulation of *ITGB1* expression by *miR-29* family members in HNSCC cells (**A**) Expression levels of *ITGB1* mRNAs 72 h after transfection of cells with 10 nM *miR-29a*, *miR-29b*, and *miR-29c*. *GUSB* was used as an internal control. ^*^P < 0.0001. (**B**) Protein expression of ITGB1 72 h after transfection with *miR-29a*, *miR-29b*, and *miR-29c*. GAPDH was used as a loading control. (**C**) *miR-29* family binding sites in the 3′UTR of *ITGB1* mRNA. Dual Luciferase reporter assays using vectors encoding putative *miR-29* family target sites in the *ITGB1* 3′UTR (positions 830-836) for wild-type and deletion constructs. Normalized data were calculated as ratios of *Renilla*/firefly luciferase activities. ^*^P < 0.0001.

We also investigated whether *ITGB1* expression was reduced by restoration of the *miR-29* family in HNSCC cells. The mRNA and protein expression levels of *ITGB1*/ITGB1 were significantly repressed in all member of miR-29 family (*miR-29a*, *miR-29b* and *miR-29c*) transfectants compared with that in mock transfectants (Figures 3A and 3B). The synergistic effects of *miR-29a, miR-29b* and *miR-29c* were evaluated the mRNA expression levels of *ITGB1* with co-transfection of *miR-29a, miR-29b* and *miR-29c* in SAS cells. However, no synergistic effects were observed (Supplementary Figure 4).

Furthermore, we performed luciferase reporter assays in SAS cells to determine whether *ITGB1* mRNA contained target sites for the *miR-29* family. We used vectors encoding the partial wild-type or deletion-type sequences of the 3′UTR of *ITGB1* with *miR-29* family target sites. Luminescence intensity was significantly reduced by co-transfection with the *miR-29* family and the vector carrying the wild-type 3′UTR of *ITGB1* mRNA (Figure 3C).

### Effects of *ITGB1* knockdown on cell proliferation, migration, and invasion in HNSCC cell lines

**Figure 4:**
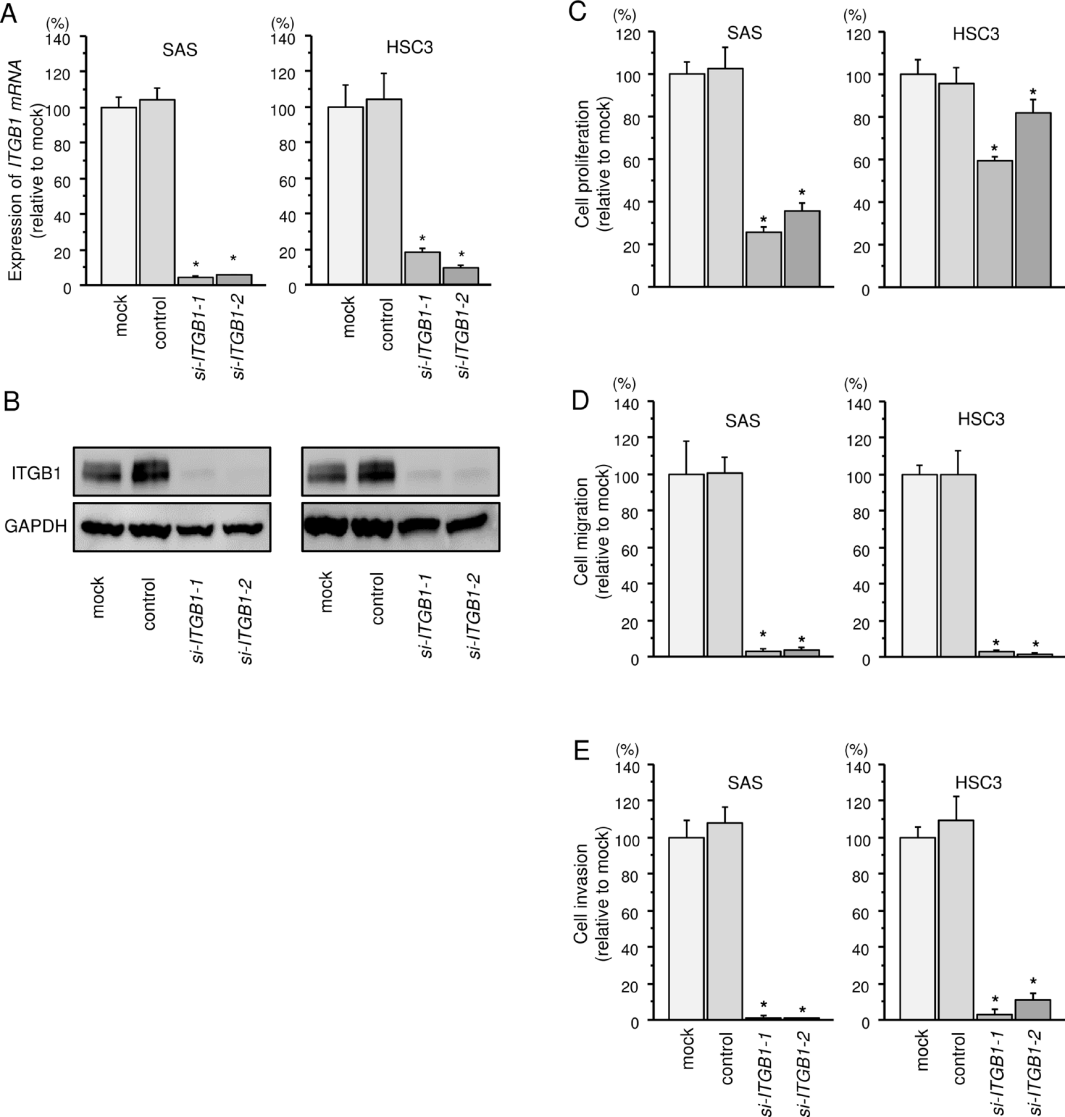
Effects of *ITGB1* silencing by siRNA transfection in HNSCC cells (**A**) *ITGB1* mRNA expression 72 h after transfection of HNSCC cells with 10 nM siRNA. *GUSB* was used as an internal control. ^*^P < 0.0001. (**B**) ITGB1 protein expression 72 h after transfection with siRNA. GAPDH was used as a loading control. (**C**) Cell proliferation was determined by XTT assays 72 h after transfection with siRNA. ^*^P < 0.0001. (**D**) Cell movement was assessed by migration assays 48 h after transfection with siRNA. ^*^P < 0.0001. (**E**) Characterization of invasion 48 h after transfection with siRNA. ^*^P < 0.0001.

Knockdown efficiency of si*-ITGB1* transfection were evaluated in SAS and HSC3 cells, and these siRNAs effectively suppressed *ITGB1*/ITGB1 expression (Figure 4A and 4B).

Cell proliferation, migration and invasion abilities were significantly inhibited in *si-ITGB1* transfectants compared with that in mock transfectants (Figure 4C–4E).

### Effects of co-transfection with *ITGB1* and the *miR-29* family in SAS cells

We performed gain-of-function analyses by ITGB1 expression vector transfection into SAS cells (Supplementary Figure 5A). Cancer cell migration and invasion abilities were enhanced by overexpression of ITGB1 in SAS cells (Supplementary Figure 5B and 5C).

**Figure 5:**
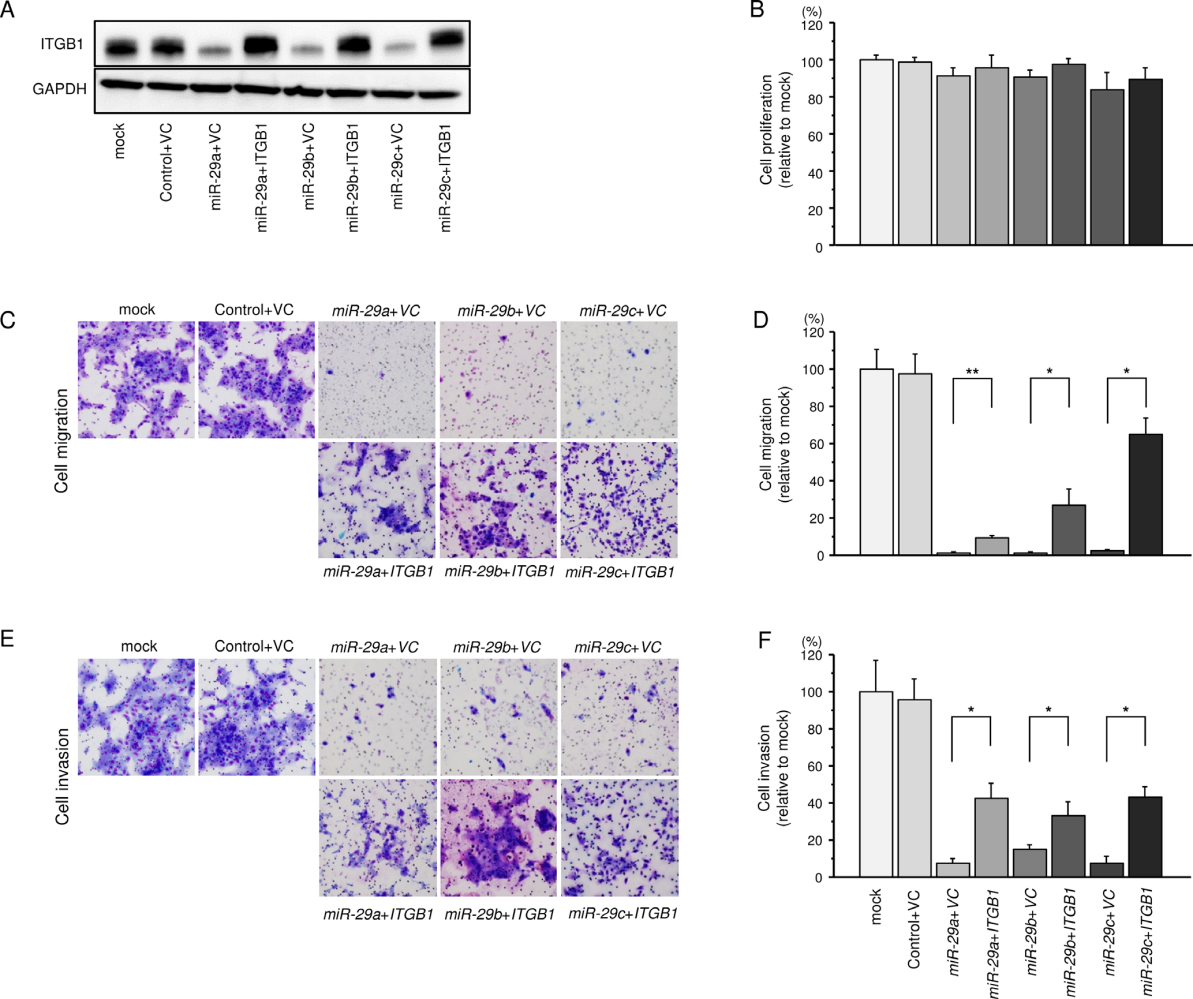
Effects of cotransfection with *ITGB1/miR-29* family on the proliferlation, migration and invasion abilities of SAS cells (**A**) Expression of ITGB1 was confirmed by western blotting 48 h after reverse transfection with the *miR-29* family and 24 h after forward transfection with the vector control (VC) and ITGB1 expression vector (1 μg) in SAS cells. GAPDH was used as a loading control. (**B**) Cell proliferation was determined 48 h after transfection with *miR-29* family members and 24 h after forward transfection with the ITGB1 expression vector (1 μg). (**C**) Phase micrographs of SAS cells in migration assays. (100× magnification). (**D**) Cell migration activity was assessed 48 h after reverse transfection with the *miR-29* family and 24 h after forward transfection with the ITGB1 expression vector (1μg). ^*^P < 0.0001, ^*^P < 0.02. (**E**) Phase micrographs of SAS cells in invasion assays. (100× magnification) (**F**). Cell invasion activity was assessed 48 h after reverse transfection with *miR-29* family members and 24 h after forward transfection with the ITGB1 expression vector (1 μg). ^*^P < 0.0001.

To validate whether the *ITGB1/miR-29* family axis was critical for the progression of HNSCC, we performed *ITGB1* rescue experiments by co-transfection with *ITGB1* and the *miR-29* family in SAS cells (Figure 5A). The results showed that the proliferation abilities of SAS cells were not recovered by *ITGB1* and *miR-29* family transfection compared with cells showing restoration of each *miR-29* family member alone (Figure 5B). In contrast, the migration and invasion abilities of SAS cells were recovered markedly by *ITGB1* and *miR-29* family transfection compared with cells showing restoration of each *miR-29* family member alone (Figure 5C–5F). These findings suggested that *ITGB1* was involved in cancer cell migration and invasion in HNSCC cells.

### Effects of *ITGB1* knockdown on downstream signalling

**Figure 6:**
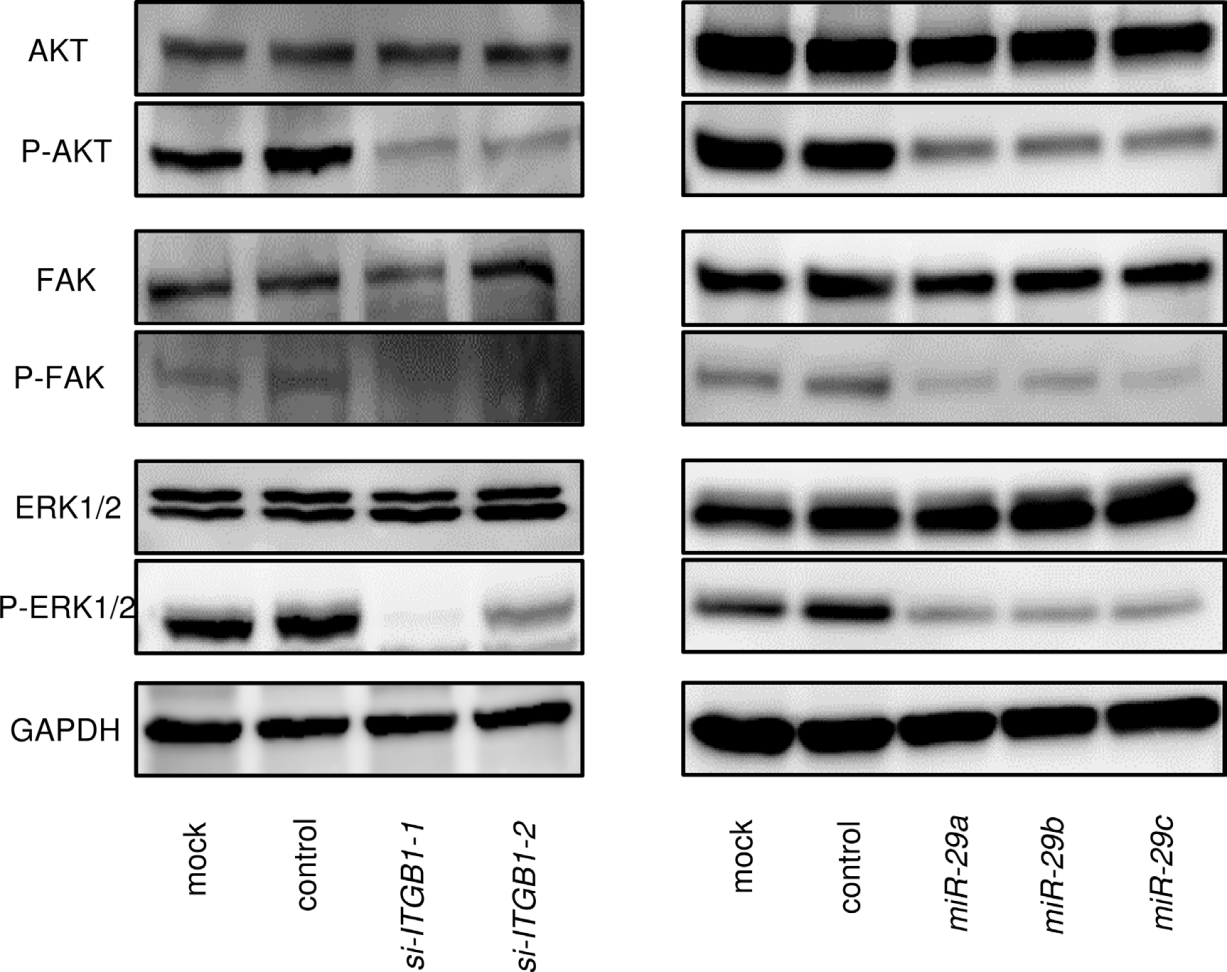
Effects of ITGB1-mediated downstream signalling Knockdown of ITGB1 and restoration of the *miR-29* family in SAS cells reduced the phosphorylation of AKT, ERK1/2, and FAK. GAPDH was used as a loading control.

We analysed the effects of downstream oncogenic signalling of *ITGB1* using siRNA transfection of SAS cells. The phosphorylation statuses of AKT (Ser473), ERK1/2 (Thr202/Tyr204), and FAK (Tyr397) were examined. Knockdown of *ITGB1* reduced the phosphorylation of AKT, ERK1/2, and FAK in SAS cells (Figure 6). We also investigated whether the expression of the *miR-29* family affected downstream signalling. Restoration of the *miR-29* family reduced the phosphorylation of AKT, ERK1/2, and FAK in SAS cells (Figure 6).

## DISCUSSION

The overall survival of patients with HNSCC recurrence or metastasis is very poor [1, 2]. Currently developed EGFR inhibitors, including monoclonal antibodies and tyrosine kinase inhibitors, have achieved only modest success in the treatment of this disease [3, 4]. Screening for novel therapeutic targets is indispensable for the development of new therapeutic strategies for patients with HNSCC recurrence or metastasis.

Our recent studies showed that antitumor miRNAs, i.e., the *miR-29* family, *miR-218*, *miR-150s*, and the *miR-199* family, significantly inhibited cancer cell migration and invasion, suggesting that these miRNAs controlled metastatic genes and pathways in HNSCC cells [9, 15, 19, 25]. To date, we have shown that several ECM-related genes, including *LAMA3*, *LAMB3*, *LAMC2*, *ITGA3*, and *ITGA6*, are overexpressed in HNSCC cells and that these genes are regulated by antitumor miRNAs [9, 15, 19, 25]. Laminins are heterotrimers containing α, β, and γ chains [26]. Laminin-332 (LAMA3, LAMB3, and LAMC2), a large multidomain molecule involved in cell adhesion and matrix assembly, is a prominent component of the ECM in SCC. Aberrantly expressed laminin-332 is correlated with patient prognosis in HNSCC [27, 28].

**Figure 7:**
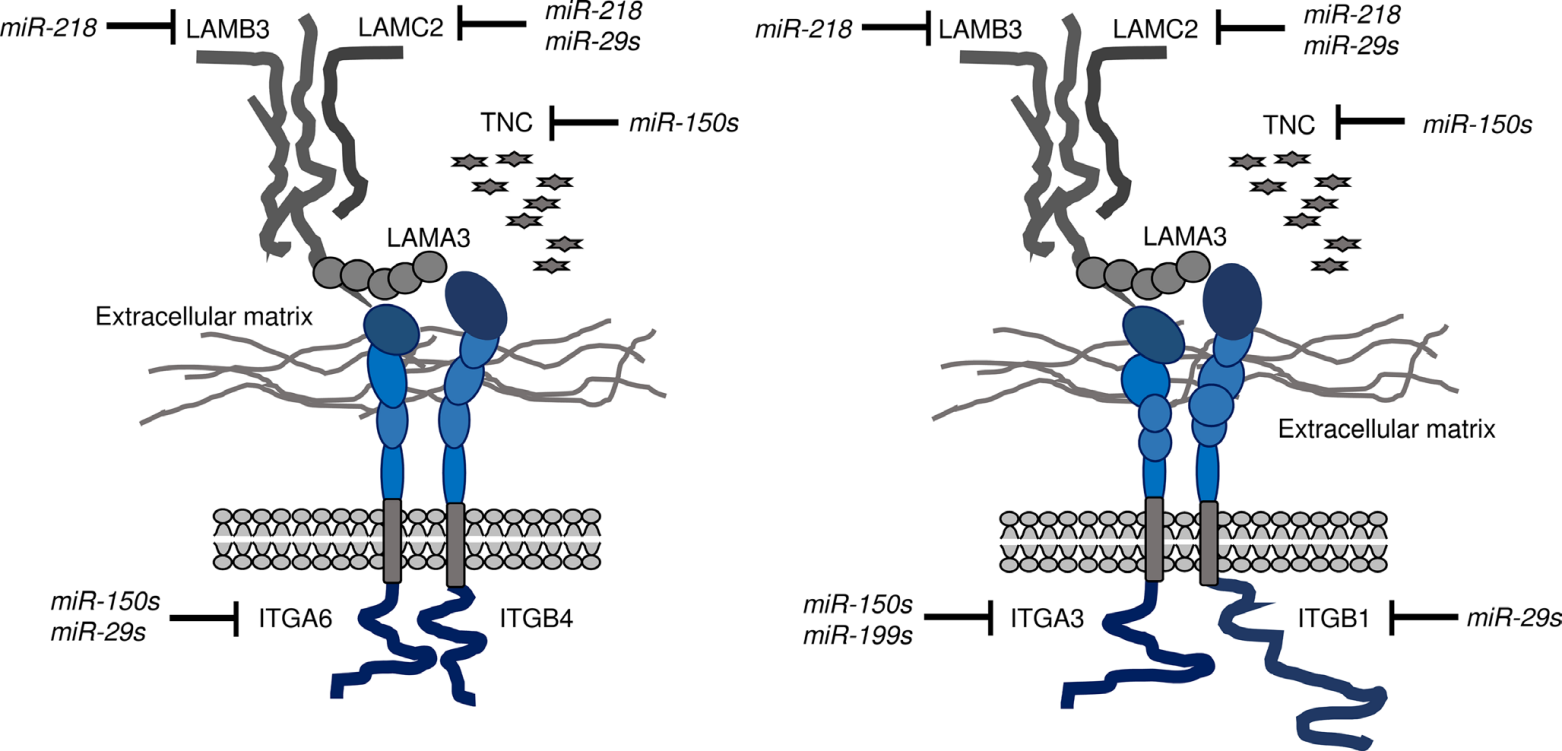
Illustration of inhibition of integrin receptors and ligands, i.e., laminin-332 and Tenascin C, by antitumor miRNAs in HNSCC cells Data from our previous studies and the present study on integrins (ITGA6/ITGB4, ITGA3/ITGB1) regulated by antitumor miRNAs (the *miR-29* family, *miR-218*, *miR-150-5p*, and *miR-150-3p*) and related ligands (*LAMA3*, *LAMB3*, *LAMC2*, and *TNC*) are summarized.

Integrins, cell adhesion molecules whose main function is to mediate mutual adhesion between cells and the ECM, e.g., laminin, collagen, elastin, and fibronectin [29, 30], belong to a large family of αβ heterodimeric transmembrane proteins; 24 heterodimeric combinations have been reported in humans [29, 30]. Laminin-332 can interact with two types of integrins, ITGA6/ITGB4 (α6β4) and ITGA3/ITGB1 (α3β1), and activation of laminin-332-mediated integrin signalling enhances cancer cell development and metastasis [26, 31]. Our present study showed that all members of the *miR-29* family directly regulated *ITGB1* in HNSCC cells. Moreover, ectopic expression of the *miR-29* family and knockdown of *ITGB1* suppressed cancer cell aggressiveness by inhibiting *ITGB1*-mediated downstream signalling. To summarise our miRNA studies to date, laminin-332-ITGA6/ITGB4 and laminin-332-ITGA3/B1 pathways were regulated by antitumor miRNAs, i.e., the *miR-29* family, *miR-218*, *miR-150-5p*/*-3p*, and the *miR-199* family, in HNSCC cells (Figure 7).

Epigenetic regulation of *ITGB1* has been reported in several studies [19, 32–34]. In gastric cancer, upregulation of telomerase reverse transcriptase suppresses the expression of *miR-29a* and induces the expression of *ITGB1* [32]. In another study, RNA sequencing demonstrated that *miR-29c* is downregulated in gastric cancer specimens and targets *ITGB1* [33]. More recently, the pivotal tumor suppressor p53 was shown to induce *miR-30e-5p*, which targets both *ITGA6* and *ITGB1* in colorectal cancer [35]. Our present and past studies showed that the *miR-29* family directly regulates both *ITGA6* and *ITGB1* in HNSCC cells [19]. Moreover, our recent study of the miRNA signature of HNSCC showed that the expression of *miR-30e-5p* was significantly reduced in HNSCC tissues [15]. These findings indicate that the *miR-29* family and *miR-30e-5p* function as important regulators of cancer cell migration and invasion. We also revealed that *miR-223* acted as an antitumor miRNA through targeting *ITGA3* and *ITGB1* in prostate cancer [34].

ITGB1 can form heterodimers with α subunits, and these heterodimers regulate numerous signalling pathways in both physiological and pathophysiological conditions [30]. Overexpression of ITGB1 was frequently observed several cancers, including HNSCC, resulting in activation of ITGB1-mediated downstream signalling pathways [32, 34–36]. Previous studies have shown that crosstalk of integrins and several growth factor receptors, such as EGFR and the hepatocyte growth factor receptor (c-Met), activated downstream signalling cooperatively [37, 38]. Overexpression and aberrant activation of EGFR signalling enhance proliferation, invasion, metastasis, and angiogenesis and are deeply involved in HNSCC aggressiveness [39, 40]. Currently, the EGFR inhibitor cetuximab has been approved for HNSCC as a first-line treatment in patients with recurrent or metastatic disease [41]. Importantly, EGFR inhibitors induced c-MET signal activation, resulting in resistance to EGFR-targeted therapy [42]. Several studies have demonstrated that activation of ITGB1-mediated signals induces radioresistance in HNSCC and breast cancer [43, 44]. Moreover, activation of ITGB1-mediated signals enhances chemoresistance in several cancers [45, 46]. Increasing knowledge of ITGB1 suggests that aberrant expression and activation of ITGB1-mediated cancer signalling is involved in cancer cell aggressiveness. Therefore, inhibition of ITGB1 and ITGB1-mediated cancer signalling is attractive as a new treatment strategy for cancer cells that have acquired treatment resistance.

In conclusions, analysis of oncogenes targeted by the antitumor *miR-29* family (*miR-29a*, *miR-29b*, and *miR-29c*) showed that *ITGA6* and *ITGB1* were directly regulated by these miRNAs in HNSCC. High expression of these oncogenic genes was associated with poor prognosis in patients with HNSCC. Inhibition of *ITGB1* significantly reduced cancer cell aggressiveness, indicating that *ITGB1* and *ITGB1*-mediated cancer signalling may be promising therapeutic targets for HNSCC. Identification of antitumor miRNA-mediated novel RNA networks may contribute to the development of new therapeutic strategies.

## MATERIALS AND METHODS

### Clinical specimens of patients with HNSCC and cell lines

The patients’ backgrounds and clinicopathological characteristics are summarised in Table 4.

**Table 4:** Clinical features of 22 patients with HNSCC

No.	Age	Sex	Location	T	N	M	Stage	Differentiaion	Remarks
1	64	F	Oral floor	4a	2c	0	IVA	moderate	IHC
2	73	M	Tongue	3	2b	0	IVA	poor	IHC
3	77	M	Tongue	2	2b	0	IVA	poor	
4	63	F	Oral floor	2	2b	0	IVA	Basaloid SCC	
5	59	M	Tongue	1	2a	0	IVA	moderate	
6	36	F	Tongue	3	1	0	III	moderate	
7	67	M	Tongue	3	0	0	III	moderate	IHC
8	60	F	Tongue	2	I	0	III	well	
9	66	M	Tongue	2	0	0	II	moderate	IHC
10	67	M	Tongue	2	0	0	II	poor-moderate	
11	76	F	Tongue	1	0	0	I	well	
12	69	M	Tongue	1	0	0	I	well	
13	73	F	Tongue	1	0	0	I	well	
14	64	M	Tongue	1	0	0	I	well	
15	70	M	Tongue	1	0	0	I	well	
16	38	M	Tongue	1	0	0	I	well	
17	51	M	Tongue	1	0	0	I	well	
18	34	F	Tongue	1	0	0	I	poor	
19	70	M	Tongue	1	0	0	I	moderate	
20	71	M	Tongue	1	0	0	I	well	
21	82	M	Oral floor	1	0	0	I	well	
22	81	M	Tongue	1	0	0	I	extremely well	

Informed consent was done properly for all patients in this study. This study protocol was approved by the Institutional Review Board of Chiba University. Two human HNSCC cell lines, SAS and HSC3, were investigated in this study.

### Expression analyses of patients with HNSCC and cell lines

PCR quantification was carried out essentially as previously described [9, 15]. To quantify the expression level of miRNAs, we utilised stem-loop qRT-PCR for *miR-29a* (assay ID: 002112; Applied Biosystems, Foster City, CA, USA), *miR-29b* (assay ID: 000413), and *miR-29c* (assay ID: 000587) following the manufacturer’s protocol. TaqMan probes and primers were used for *ITGB1* expression (assay ID: Hs00559595_m1; Applied Biosystems). *GUSB* (assay ID: Hs99999908_m1; Applied Biosystems) and *RNU48* (assay ID: 001006; Applied Biosystems) were used as internal controls.

### Transfection of HNSCC cell lines with miRNA mimic, small interfering RNA (siRNA), and plasmid vector

Pre-miR miRNA precursors (*miR-29a-3p*; P/N: MC12499, *miR-29b-3p*; P/N: MC10103, *miR-29c-3p*; P/N: MC10518, and negative control miR; P/N: AM17111; Applied Biosystems) were used in gain-of-function assays. si-*ITGB1* (P/N: HSS105559 and HSS105560; Invitrogen) was used in loss-of-function assays. Transfection procedures of RNAs or plasmid DNA were described as previously [9, 15].

### Cell proliferation, migration, and invasion assays

Cell proliferation, migration, and invasion assays were carried out as previously described [9, 15].

### Identification of putative genes regulated by the *miR-29* family in HNSCC cells

Oncogenic genes regulated by the *miR-29* family were identified by a combination of *in silico* database analyses and gene expression analyses as described previously [9, 15]. The microarray data were deposited into GEO database (http://www.ncbi.nlm.nih.gov/geo/; accession number: GSE47657).

### Western blotting

Immunoblotting was performed with rabbit anti-ITGB1 antibodies (1:1000, #9699; Cell Signaling Technology, Danvers, MA, USA), anti-Akt antibodies (1:1000, #4691; Cell Signaling Technology), anti-phospho-Akt antibodies (1:1000, #4060; Cell Signaling Technology), anti-extracellular signal-regulated kinase (ERK1/2) antibodies (1:1000, #4695; Cell Signaling Technology), anti-phospho-ERK1/2 antibodies (1:2000, #4370; Cell Signaling Technology), anti-focal adhesion kinase (FAK) antibodies (1:1000, #3285; Cell Signaling Technology), and anti-phospho-FAK antibodies (1:1000, #8556; Cell Signaling Technology). Anti-GAPDH antibodies (1:10000, ab8246; Abcam, Cambridge, UK) were used as an internal control.

### Immunohistochemistry

Anti-ITGB1 antibodies (#9699; Cell Signaling Technology) were used in immunohistochemistry.

### Plasmid construction and dual-luciferase reporter assays

The wild-type or deletion-type sequences of the 3′-untranslated region (UTR) of *ITGB1* in *miR-29a*, *miR-29b*, and *miR-29c* target sites were inserted in the psiCHECK-2 vector (C8021; Promega, Madison, WI, USA). The procedure for dual luciferase reporter assays was described previously [9].

### Analysis of HNSCC specimens using The Cancer Genome Atlas (TCGA)

HNSCC specimens in TCGA database (https://tcga-data.nci.nih.gov/tcga/) were divided into two groups, i.e., high and low expression of *ITGB1.* The groups were analysed by Kaplan–Meier survival curves and log-rank statistics using OncoLnc (http://www.oncolnc.org) [47]. The genomic and clinical data were retrieved from cBioportal (http://www.cbioportal.org/), which were downloaded on May 9, 2017. Detailed information on the method is described in our previous paper [48].

### Statistical analysis

Statistical analysis was performed as described previously [9, 15]. All analyses were performed using Expert StatView (version 4, SAS Institute Inc., Cary, NC, USA). Multivariate Cox proportional hazard regression models were used to determine independent factors for survival with JMP Pro 13.

## SUPPLEMENTARY MATERIALS FIGURES


