# Supplementary material for: Inhibition of integrin β1-mediated oncogenic signalling by the antitumor microRNA-29 family in head and neck squamous cell carcinoma
Source: Oncotarget. 2017 Dec 11;9(3):3663–76. doi: 10.18632/oncotarget.23194 (PMC5790491; doi:10.18632/oncotarget.23194)
Supplement: Supplementary file 1 [file oncotarget-09-3663-s001.pdf]

Inhibition of integrin β1-mediated oncogenic signalling by the antitumor *microRNA-29* family in head and neck squamous cell carcinoma

SUPPLEMENTARY MATERIALS

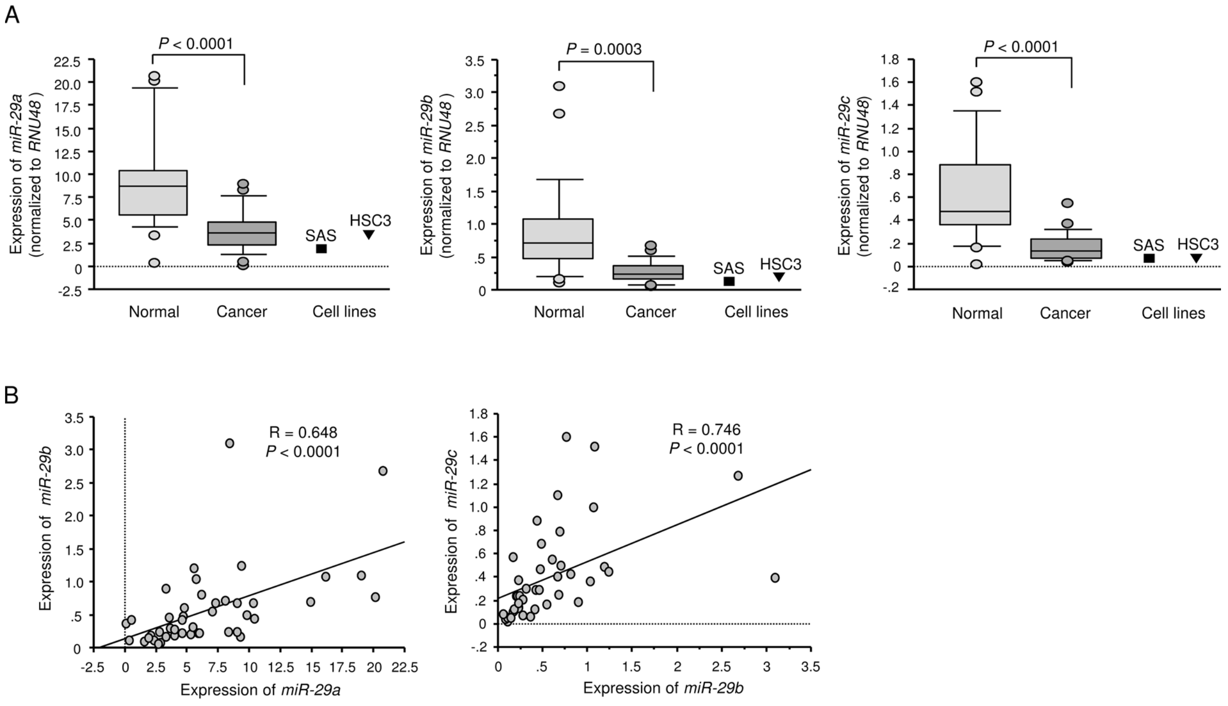

Supplementary Figure 1: Expression levels of *miR-29* family members in HNSCC clinical specimens and cell lines. (**A**) Expression levels of *miR-29a, miR-29b*, and *miR-29c* in HNSCC clinical specimens and cell lines. *RNU48* was used as an internal control. (**B**) The positive correlations between *miR-29a and miR-29b* and between *miR-29b* and *miR-29c* expression. Spearman's rank test was used to evaluate the correlation.

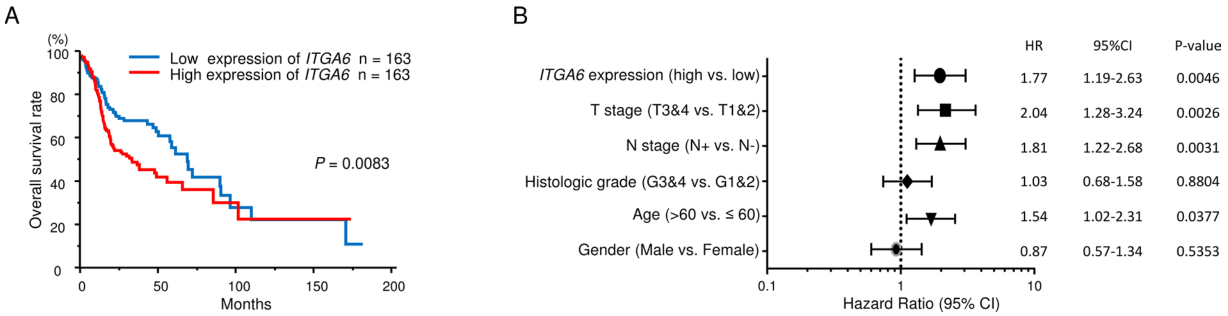

Supplementary Figure 2: Associations between *ITGA6* expression levels and clinical parameters in patients with HNSCC. (**A**) Kaplan–Meier survival curves and (**B**) hazard ratios (HRs), as determined using data from TCGA database.

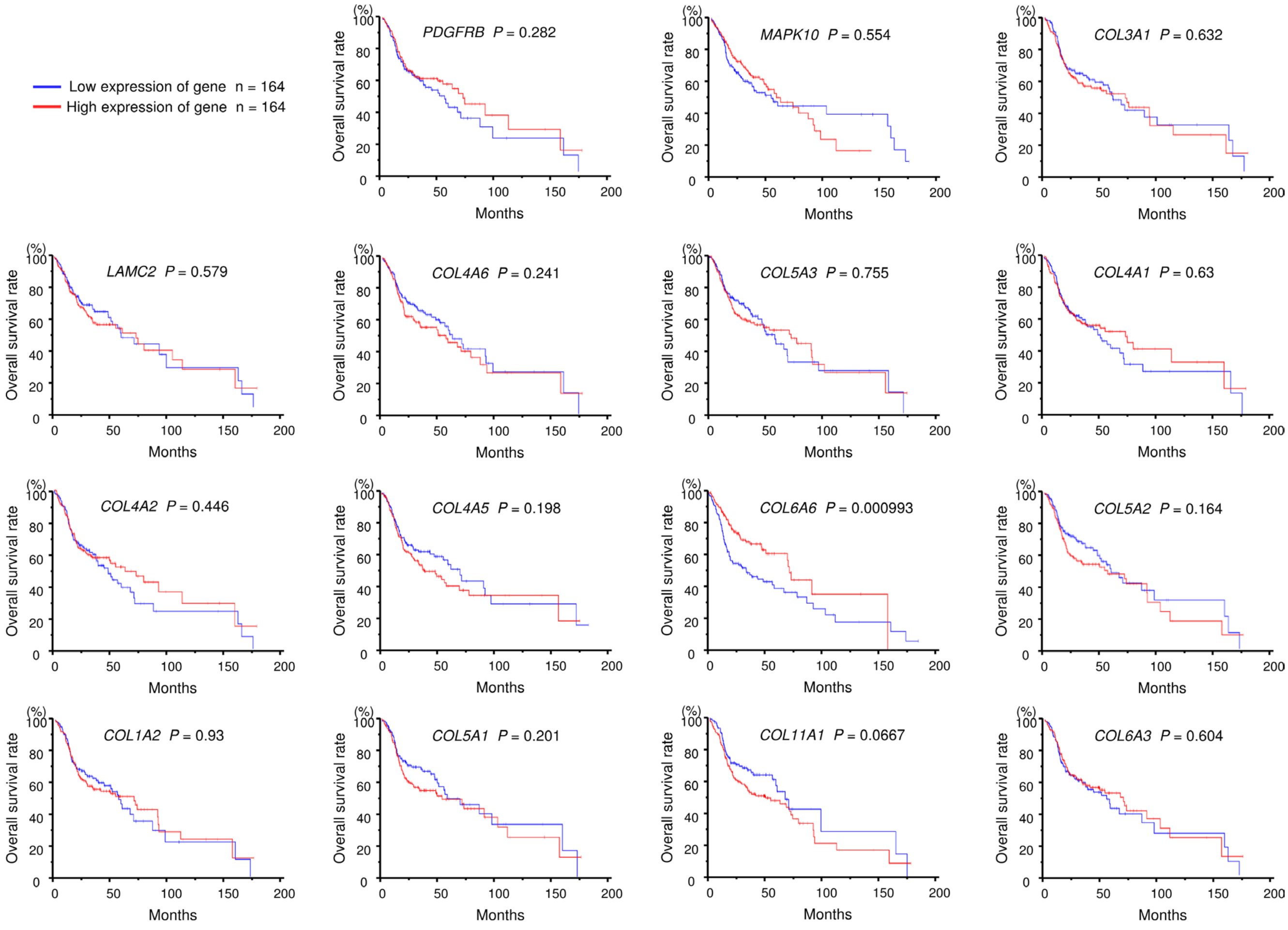

Supplementary Figure 3: Associations between expression levels and overall survival in patients with HNSCC. Kaplan-Meier plots of overall survival with log-rank tests for 15 genes (Tables 2 and 3) with high and low expression in patients with HNSCC using TCGA database.

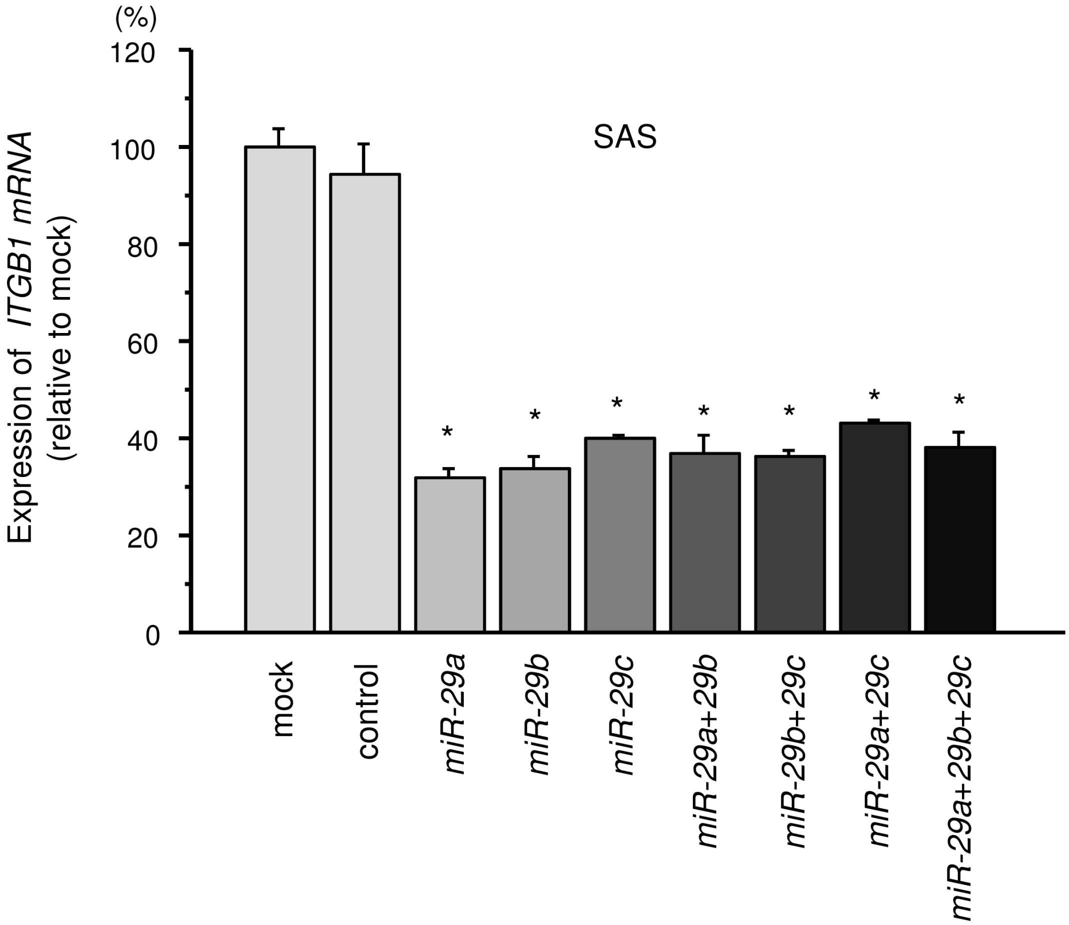

Supplementary Figure 4: The synergistic effects of *miR-29a, miR-29b* and *miR-29c* on SAS cells. Expression levels of *ITGB1* mRNAs 72 h after transfection of cells with *miR-29a*, *miR-29b*, and *miR-29c*. *GUSB* was used as an internal control. $^{*}P < 0.0001$.

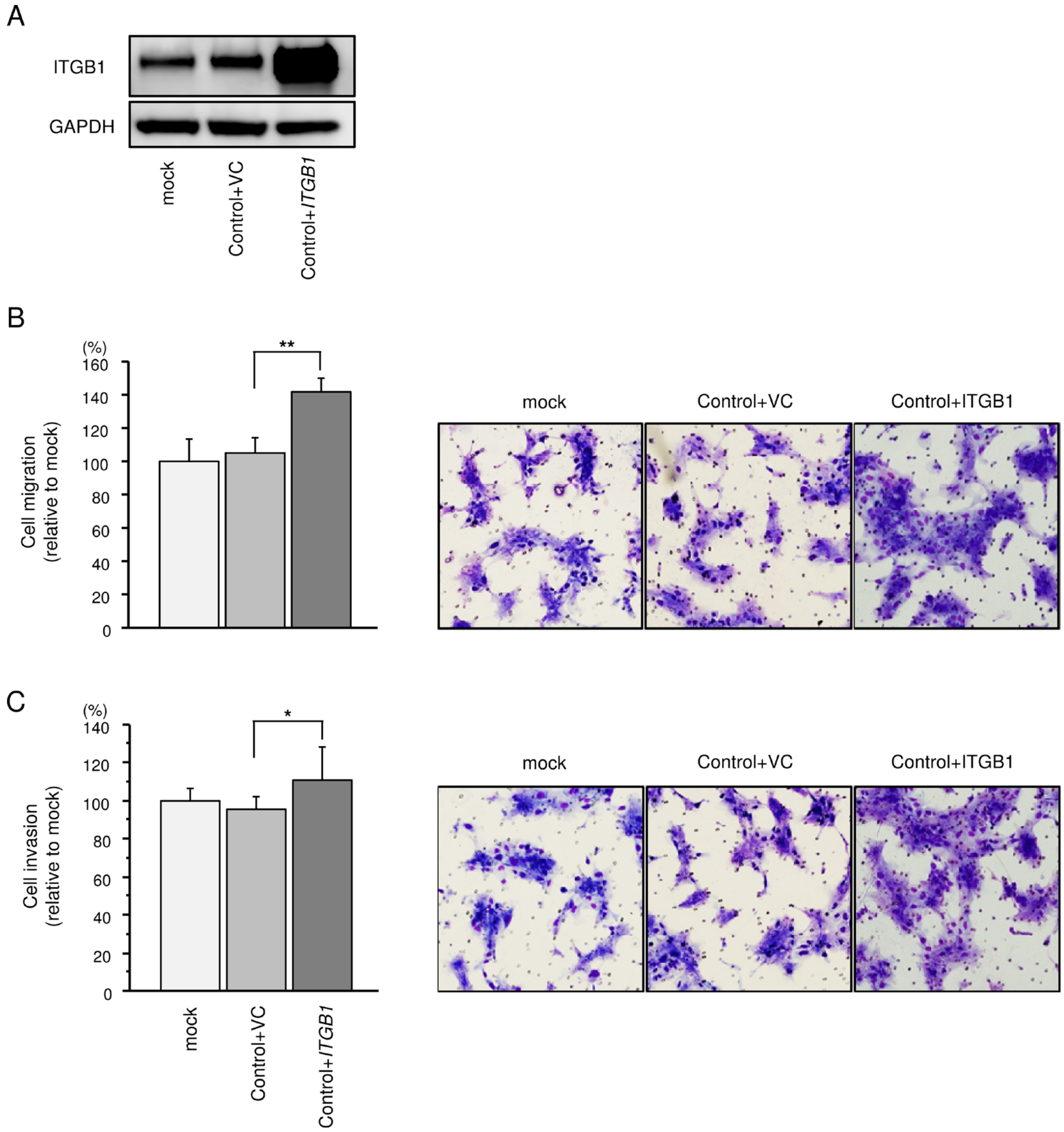

Supplementary Figure 5: Gain-of-function analyses by ITGB1 expression vector transfection into SAS cells. (**A**) Overexpression of ITGB1 was confirmed by western blotting 48 h after reverse transfection with the microRNA-control and 24 h after forward transfection with the vector control (VC) and ITGB1 expression vector (1μg) in SAS cells. GAPDH was used as a loading control. (**B**, **C**) Cancer cell migration and invasion abilities were enhanced by overexpression of ITGB1 in SAS cells. $^{*}P < 0.05$. $^{**}P < 0.0001$. Analyses were assessed 48 h after reverse transfection with the microRNA-control and 24 h after forward transfection with the ITGB1 expression vector (1 μg).
